# High expression of P4HA3 in obesity: a potential therapeutic target for type 2 diabetes

**DOI:** 10.1590/1414-431X2022e11741

**Published:** 2022-08-15

**Authors:** Langen Zhuang, Can Li, Xiaolei Hu, Qingqing Yang, Xiaoyan Pei, Guoxi Jin

**Affiliations:** 1 Department of Endocrinology, The First Affiliated Hospital of Bengbu Medical College, Bengbu, Anhui, China; 2 Shangyi Health Check-up Centre, Zibo, Shandong, China

**Keywords:** P4HA3, Obesity, T2DM

## Abstract

The aims of the present study were to evaluate the expression of prolyl 4-hydroxylase subunit alpha 3 (*P4HA3*) in adipocytes and adipose tissue and to explore its effect on obesity and type 2 diabetes mellitus (T2DM). We initially demonstrated that *P4HA3* was significantly upregulated in the subcutaneous adipose tissue of obesity and T2DM patients, and its functional roles in adipocyte differentiation and insulin resistance were investigated using *in vitro* and *in vivo* models. The knockdown of *P4HA3* inhibited adipocyte differentiation and improved insulin resistance in 3T3-L1 cells. In C57BL/6J db/db mice fed with a high fat diet (HFD), silencing *P4HA3* significantly decreased fasting blood glucose and triglycerides (TG) levels, with concomitant decrease of body weight and adipose tissue weight. Further analysis showed that *P4HA3* knockdown was correlated with the augmented IRS-1/PI3K/Akt/FoxO1 signaling pathway in the adipose and hepatic tissues of obese mice, which could improve hepatic glucose homeostasis and steatosis of mice. Together, our study suggested that the dysregulation of *P4HA3* may contribute to the development of obesity and T2DM.

## Introduction

Type 2 diabetes mellitus (T2DM) is one of the most prevalent metabolic disorders, which is characterized by insulin resistance (1). Obesity is a state of excessive fat accumulation in the adipose tissue and is a major risk factor for many metabolic diseases such as dyslipidemia, insulin resistance, and T2DM (2). Although most individuals diagnosed with T2DM are obese, not all obese individuals will develop T2DM (3). A growing number of studies have investigated differential gene expression profiles between obese and lean individuals (4,5). However, genes implicated in obesity predisposition are not necessarily associated with T2DM (6,7). Certain genes are associated with both obesity and T2DM, which seems to increase the risk of obesity and the chance of T2DM development (6,7).

As the main component of adipose tissue, adipocytes are considered to be an important link between obesity and T2DM development due to their secretory function (8). Many cytokines synthesized and secreted by adipocytes are involved in insulin-mediated lipid metabolism and glucose homeostasis (9). The dysregulation of adipocyte cytokines not only leads to a decrease in insulin-mediated glucose uptake by influencing insulin signaling pathway, but also increases ectopic lipid accumulation, which eventually aggravates insulin resistance and T2DM (10).

Prolyl 4-hydroxylase (P4H) is a key enzyme in collagen biosynthesis, which contains three catalytic subunits (P4HA1, P4HA2, and P4HA3) (11). Recently, several studies have suggested P4H as an oncogene in multiple tumors, since its high expression level is positively correlated with tumor growth and poor survival. The high expression level of P4HA1 is associated with the advanced degree of malignancy of glioma cells (12). Silencing P4HA2 can inhibit the invasion of breast tumor cells (13). The other subunit P4HA3 has been reported to be significantly upregulated in gastric cancer tissue compared with normal gastric tissue (14). In addition, *P4HA3* gene is highly expressed in breast cancer tissue and elevated *P4HA3* expression is correlated with poor survival outcomes (15). However, whether P4H subunits are implicated in the progression of metabolic diseases is largely unclear.

In this study, we analyzed the transcriptomic profiles of adipocytes from lean individuals, non-diabetic obese individuals, and obese individuals with T2DM from a previously published microarray dataset (GSE133099). Then, we evaluated the expression of *P4HA3* in adipocytes and adipose tissue and explored its effect on obesity and T2DM.

## Material and Methods

### Clinical samples

Subcutaneous adipose tissue samples were collected from 30 lean patients (male=17; female=13), 30 obese non-diabetics patients (male=16; female=14) and 30 obese with T2DM patients (male=17; female=13) at the First Affiliated Hospital of Bengbu Medical College between June 2019 and July 2020. The specimens were immediately frozen with liquid nitrogen and stored in a −80°C freezer. The diagnosis of obesity was based on the China National Nutrition and Health Survey (CNNHS) data: a BMI of ≥28 kg/m^2^ in Chinese adults was defined as obesity. This study was approved by the Institutional Ethics Review Board of the First Affiliated Hospital of Bengbu Medical College.

### Animals

C57BL/6J db/db mice are mice in “C57 black 6 genetic background” with db/db mutation (genetic mutation with defective leptin receptor), which develop obesity and T2DM. C57BL/6J db/db female mice (2 months old) were purchased from the Animal Research Center of Nanjing University (China) and kept in a pathogen-free facility and maintained under a 12-h light/dark cycle at 22°C. Mice were fed with a high-fat diet (HFD) (19% protein, 36% carbohydrate, and 45% fat; Harlan-Teklad TD.06415, USA). At the 10th week of feeding, mice were randomly divided into si-NC group and si-P4HA3 group. sh-P4HA3 group mice were intraperitoneally injected with adeno-associated virus carrying P4HA3 shRNA (AAV-shRNA-P4HA3), while sh-NC group mice were injected with AAV-scrambled shRNA. At the 20th week of feeding, the mice were sacrificed after 4-h fasting. Animal care and experimental procedures were approved by the Ethics Committee in Animal Experimentation of the First Affiliated Hospital of Bengbu Medical College (China).

### Adeno-associated virus preparation and injection

AAV9 virus containing shRNA targeting P4HA3 was produced by SunBio (China), with scramble non-targeting shRNA used as control. For virus injection, mice were anesthetized with isoflurane (1-4%), and placed in a prone position. The virus was diluted in sterile PBS (1×10^12^ vg/mL) and injections were administered intraperitoneally using an insulin syringe. Primers binding within the AAV inverse terminal repeats (ITRs) were used to measure the virus titer with quantitative polymerase chain reaction (qPCR) as in a previous study (16).

### Cell transfection

The 3T3-L1 adipocytes (5×10^4^ cells/well) were seeded into 6-well plates and cultured at 37°C in a humidified incubator with 5% CO_2_. Then, the cells were transfected with 100 nM siRNA targeting P4HA3 (Shanghai GenePharma Co., Ltd, China) using Lipofectamine^®^ 3000 (Invitrogen; Thermo Fisher Scientific, Inc., USA) according to the manufacturer's protocol. The cells transfected with scramble siRNA (si-NC) were used as control.

### Glucose and insulin tests

Glucose and insulin tests were performed by intraperitoneally injecting glucose (2 g/kg, 20% wt/vol d-glucose [Sigma, USA] in 0.9% wt/vol saline) or insulin (0.75 unit/kg in 0.9% wt/vol saline) in mice that had been fasted for 6 h. Blood glucose levels were measured at 0, 20, 40, 80, and 120 min using an Infinity glucose meter (US Diagnostics, USA).

### Body mass and composition measurements

Mice were weighed on an electronic scale every 1 week. Body composition was determined by time domain-nuclear magnetic resonance (TD-NMR) on a Minispec Analyst AD lean/fat analyzer (Bruker Optics, Germany).

### Preadipocyte isolation and adipocyte differentiation

Preadipocytes from human abdominal subcutaneous adipose tissue (SAT) were isolated and cultured following standard protocols. In brief, to obtain stromal cells, SAT was digested with collagenase and separated from mature adipocytes by centrifugation at 500 *g* for 15 min at 4°C. The remaining cells were incubated in erythrocyte lysis buffer for 10 min at room temperature to eliminate red blood cells. Cell debris was removed by filtering the cell suspension through a 70-μm nylon filter. After centrifugation, the pelleted preadipocytes were plated in basal medium consisting of DMEM/F-12 (Gibco, USA) supplemented with 10% fetal calf serum (FCS) and incubated for 16-18 h. After incubation, cells were detached by trypsin and counted. Cells were seeded in a 6-well plate at a density of 1×10^5^ cells per cm^2^ and cultured in complete medium until 70% confluence. To initiate differentiation, the culture medium was replaced with 2 mL MDI induction medium per well (0.5 mM methylisobutylxanthine, 1 µM dexamethasone, 10 µg/mL insulin in DMEM medium). On day 3 of differentiation, MDI induction medium was replaced with 2 mL insulin medium (DMEM containing 10 µg/mL insulin). On Day 6, insulin medium was replaced with fresh DMEM. On day 10, fully differentiated adipocyte-like cells were obtained for subsequent analysis.

For 3T3-L1 adipocyte differentiation, cells were seeded in a 6-well plate at a density of 5×10^5^ cells/well. After 24 h, the culture medium was replaced with 3 mL MDI induction medium per well. The medium was changed every 2 days. On Day 7, the differentiated adipocyte-like cells were subject to further analysis.

### Oil Red O staining

Mature adipocytes were determined by Oil Red O (ORO) staining. After washing with PBS, the cells were fixed with ice-cold acetone for 30 min. Fixed cells were washed with PBS and stained with 30% ORO in isopropanol for 60 min to visualize intracellular lipid deposits. For quantification, ORO-stained particles were eluted with 100% isopropanol and analyzed using Thermo Scientific Varioskan Flash (USA) for spectrophotometry readings at 514 nm.

### Western blot analysis

Radioimmunoprecipitation assay buffer (RIPA) (Sangon, China) was used to extract proteins from cultured cells and the concentration was determined using a bicinchoninic acid kit (Sangon Biotech Co., Ltd.). Total protein (10 µg) was resolved by 12% sodium dodecyl sulphate-polyacrylamide gel electrophoresis (SDS-PAGE). The proteins were transferred to polyvinylidene difluoride (PVDF) membranes. Non-fat milk (10%) in PBS buffer (90%) was used to block the membranes at room temperature for 1 h. Then, the membranes were incubated with primary antibody at 4°C overnight and secondary antibody at room temperature for 2 h. The immunoreactive bands were developed using an enhanced chemiluminescence kit (Santa Cruz, USA, sc-2048) and photographed on a gel imager system (Bio-Rad, USA). The densitometry analysis was performed with ImageJ software (IBM, USA). The antibodies used in this study were as follows (all from Abcam, USA): GAPDH (1:2500; ab9485; USA); C/EBP-α (1:1000; ab40761); PPAR-γ (1:1000; ab178860); C/EBP-α (1:1000; ab40761;); IRS (1:10000; ab40777); p-IRS (1:10000; ab109543); AKT (1:500; ab8805); p-AKT (1:500; ab38449); FoxO1 (1:1000; ab179450); p-FoxO1 (1:1000; ab259337); PI3K (1:1000; ab191606); p-PI3K (1:1000; ab278545); β-actin (1:1000; ab8226). In addition, HRP-linked secondary antibody was used (1:3000; Cell Signaling #7074, USA).

### Real-time RT-PCR

Trizol reagent (Thermo Fisher Scientific, 15596026) was used to extract total RNA according to the instructions. The purified total RNA was dissolved in DEPC water and its concentration was measured with NanoDorp. One microgram of total RNA was used for reverse-transcription using the cDNA PrimeScript™ RT reagent Kit (TaKaRa, China). Quantitative expression analysis was performed using SYBR premix EX TAQ II kit (RR820A, Takara) on the Roche LightCycler 480 qPCR system (Roche, Germany). The PCR cycling condition used was: 95°C for 5 min, 40 cycles of 95°C for 30 s, 60°C for 30 s, and 72°C for 60 s. Relative gene expression level was calculated by 2^-Δ ΔCT^ method using GAPDH as the reference gene. All quantitative qPCR reactions were performed in triplicate. The primers used for real-time qPCR are shown in Supplementary Table S1.

### Glucose uptake assay

Glucose uptake in differentiated 3T3-L1 adipocytes was assessed by analysis of 2-[1,2-3H (N)]-DOG uptake. The cells were incubated with 100 nM insulin for 30 min prior to glucose uptake assay. Assays were performed in Krebs-Ringer phosphate buffer supplemented with 0.2% bovine serum albumin (downstream media). Briefly, cells were washed in downstream media and incubated in downstream media containing 5 mM glucose for 2 h. After washing with downstream media without glucose, 2-[1,2-3H (N)]-DOG (0.125 μCi/well) was added to cells for 10 min. Cells were then immediately washed 3 times with chilled PBS and lysed in PBS with 0.1% Triton-X100. An aliquot (50 μL) from the lysate was taken for protein measurement and the remainder was transferred to scintillation fluid for radioactive counting. The recorded counts per minute values were normalized to the total cellular protein level for each sample.

### Tissue and intracellular triglyceride (TG) analyses

Lipids were extracted and dissolved in chloroform. An aliquot (30 μL) was removed from each sample for quantification. Cultured cells were directly lysed in 1% Triton X-100 in PBS. After centrifugation at 500 *g* for 10 min at 4°C, a 30-μL aliquot was taken from each sample for measurement. TG were measured using Triglyceride Quantification Colorimetric/Fluorometric Kit (Sigma, mak266), according to the manufacturer's instructions.

### Hematoxylin and eosin (H&E) staining

H&E staining was performed using H&E Stain Kit (ab245880, Abcam). Deparaffinized/hydrated sections were placed in adequate Mayer's hematoxylin (Lillie's Modification) to completely cover the tissue section and incubated for 5 min. The section was rinsed twice with distilled water to remove excess stain. Then, adequate Bluing Reagent was applied to completely cover the tissue section and was then incubated for 30 s. After washing with distilled water, the section was dehydrated in absolute alcohol, followed by staining with Eosin Y Solution to completely cover the tissue for 2-3 min. The section was rinsed three times using absolute ethanol and then mounted on a slide. The images were collected under an inverse microscope (LEICA DM500, USA) at 200× magnification.

### Microarray analysis

The published microarray dataset (GSE133099) was retrieved from Gene Expression Omnibus (GEO). Differentially expressed genes were identified by the edgeR package in R software. Deferentially expressed (DE) genes were identified based on adjusted P value (<0.05) and the |log_2_FC (fold change)| ≥1.

### Statistical analyses

All statistical analyses were performed with R (version 3.6.3) and SPSS v26.0 (SPSS Inc., USA). The statistical difference between two groups was compared using unpaired Student's *t*-test and comparisons among multiple groups were analyzed using one-way analysis of variance (ANOVA) with Tukey's *post hoc* test for pairwise comparison. P value of <0.05 was considered statistically significant and all tests were two-sided.

## Results

### *P4HA3* was significantly upregulated in obese and T2DM patients.

**Figure 1:**
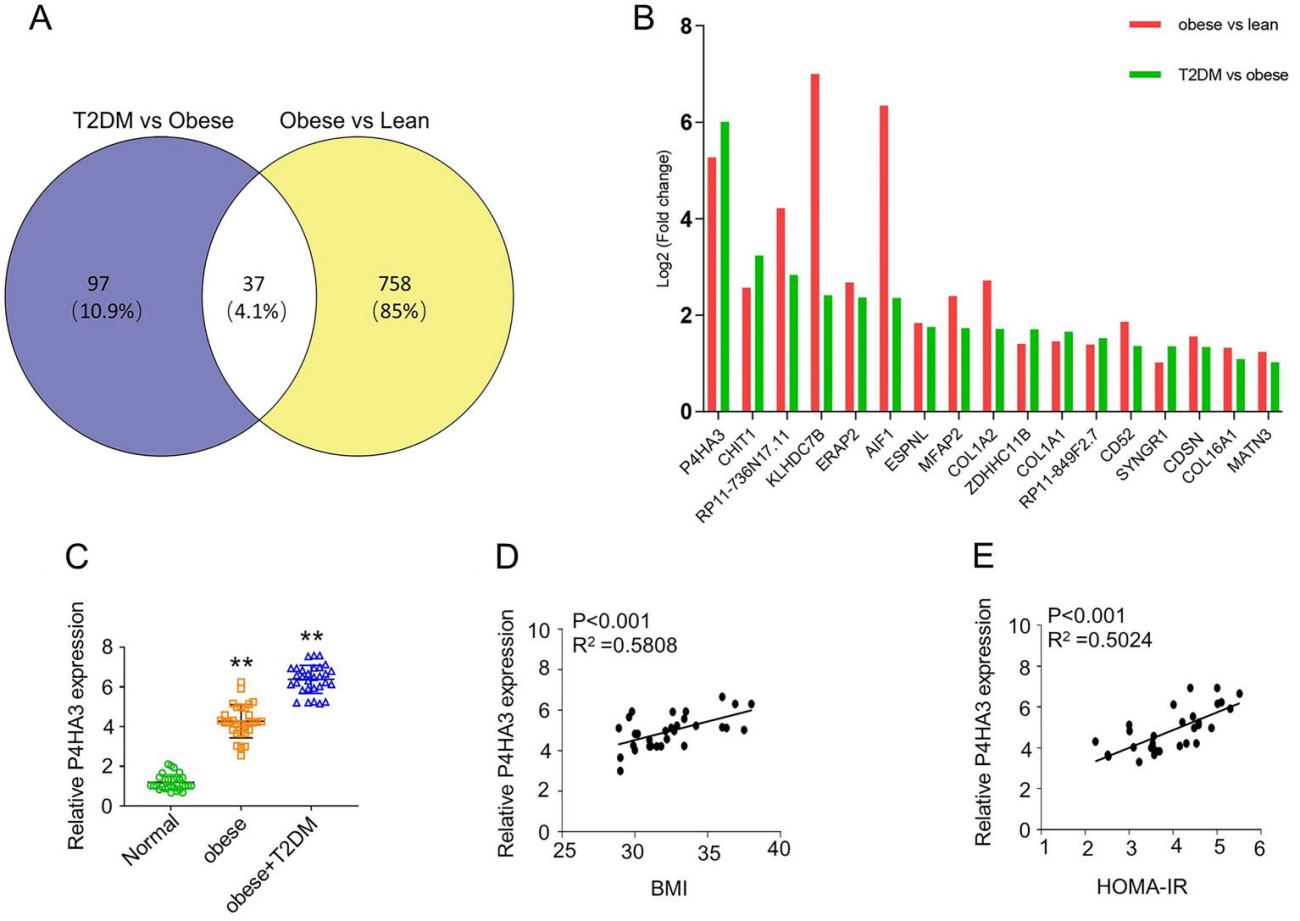
*P4HA3* was significantly upregulated in obese and type 2 diabetes mellitus (T2DM) patients. **A**, Venn diagram of the differentially expressed (DE) genes in 2 pairwise comparisons: lean obese, obese (non-diabetic) *vs* diabetic. **B**, Top ranked DE genes in both groups (|logFC| ≥1 and P<0.05), with their log_2_ fold-changes. **C**, *P4HA3* expression in adipose tissue from 30 individuals with normal body mass index (BMI), 30 obese patients, and 30 obese patients with T2DM. Pearson correlation between *P4HA3* expression level in adipose tissue and BMI in 30 obese patients (**D**) and between *P4HA3* expression level in adipose tissue and Homeostatic Model Assessment for Insulin Resistance (HOMA-IR) of 30 obese with T2DM patients (**E**). **P<0.01 (ANOVA).

Based on the published microarray dataset (GSE133099), we analyzed the expression profiles in mature adipocytes. In non-diabetic individuals, we identified 795 significantly differentially expressed (DE) genes between obese and lean subjects. In obese individuals, we identified 134 DE genes between diabetic and non-diabetic individuals. A total of 37 DE genes were shared between these two comparison groups (Figure 1A). Notably, P4HA3 was one of the most up-regulated genes in obese and diabetic individuals (Figure 1B). The expression level of *P4HA3* in adipocytes of obese individuals was much higher than that of lean individuals, which was further elevated in adipocytes of the obese individuals with T2DM.

To validate the upregulation of *P4HA3* in obese and T2DM, we collected subcutaneous fat tissue samples from 30 obese patients, 30 obese with T2DM patients, and 30 lean people. As shown in Table 1, the individuals in all groups were age-matched, and the two obese groups showed similar anthropometrical characteristics, which included body mass index (BMI), body fat (BF), waist circumference, and waist-to-hip ratio. However, these characteristics were significantly higher in the two obese groups compared with lean groups. Moreover, obese patients showed increased levels of leptin and triacylglycerols but reduced concentrations of high-density lipoprotein (HDL) cholesterol. Obese patients with T2DM exhibited higher fasting blood glucose (FBG), glycosylated hemoglobin (HbA1c), and fasting insulin level (FINS) than lean and obese non-diabetic patients.

**Table 1:** Baseline characteristics of the study population.

Factor	Normal (n=30)	Obese (n=30)	Obese+T2DM (n=30)
Age (years)	42±2.05	39±1.05	42±1.85
Male (female) (n)	17 (13)	16 (14)	17 (13)
BMI (kg/m^2^)	20.64±1.49	31.60±1.37	33.72±1.45**
BF (%)	24±0.78	30±4.46**	31±3.14**
WC (cm)	73.65±2.36	93.94±7.47**	98.09±10.39**
HC (cm)	91.46±4.32	104.58±5.65**	103.55±5.57**
WHR	0.79±0.06	0.92±0.05**	0.95±0.09**
Leptin (ng/mL)	9.28±7.31	26.7±10.4**	27.75±10.23**
TG (mM)	0.86 (0.67-1.17)	1.57 (1.25-2.16)**	1.57 (1.08-2.66)**
HDL (mM)	1.62±0.39	1.30±0.31**	1.38±0.34*
FBG (mM)	4.98±0.47	5.42±0.59	9.68±4.01^##^
HbA1c (%)	5.34±0.25	5.65±0.35	8.31±2.53^##^
FINS (μIU/mL)	5.78 (4.65-6.66)	10.52 (5.92-14.08)	12.7 (9.00-21.64)^##^

Data are reported as means±SD or median and interquartile range. *P<0.05; **P<0.01, compared with the normal group; ^##^P<0.01 compared with the obese group (ANOVA). BMI: body fat index; BF: body fat: WC: waist circumference; HC: hip circumference; WHR: waist-to-hip ratio; TG: triglycerides; HDL: high-density lipoprotein; FBG: fasting blood glucose; HbA1c: glycosylated hemoglobin; FINS: fasting insulin level.

After comparing these subcutaneous fat tissues, we found that the expression of *P4HA3* from obese patients was higher than lean people, and obese patients with T2DM showed an even higher level of *P4HA3* than obese non-diabetic patients (Figure 1C). Next, a potential correlation between *P4HA3* expression level and BMI index was examined using Pearson correlation analysis, which showed a positive correlation between P4HA3 level and BMI in 30 obese patients (Figure 1D). More importantly, increased P4HA3 expression was significantly associated with HOMA-IR (Homeostatic Model Assessment for Insulin Resistance) in obese with T2DM patients (Figure 1E). Together, these findings suggested that *P4HA3* upregulation in adipose tissues was implicated in the development of obesity and obesity-associated T2DM.

### *P4HA3* knockdown suppressed adipocyte differentiation in 3T3-L1 cells

**Figure 2:**
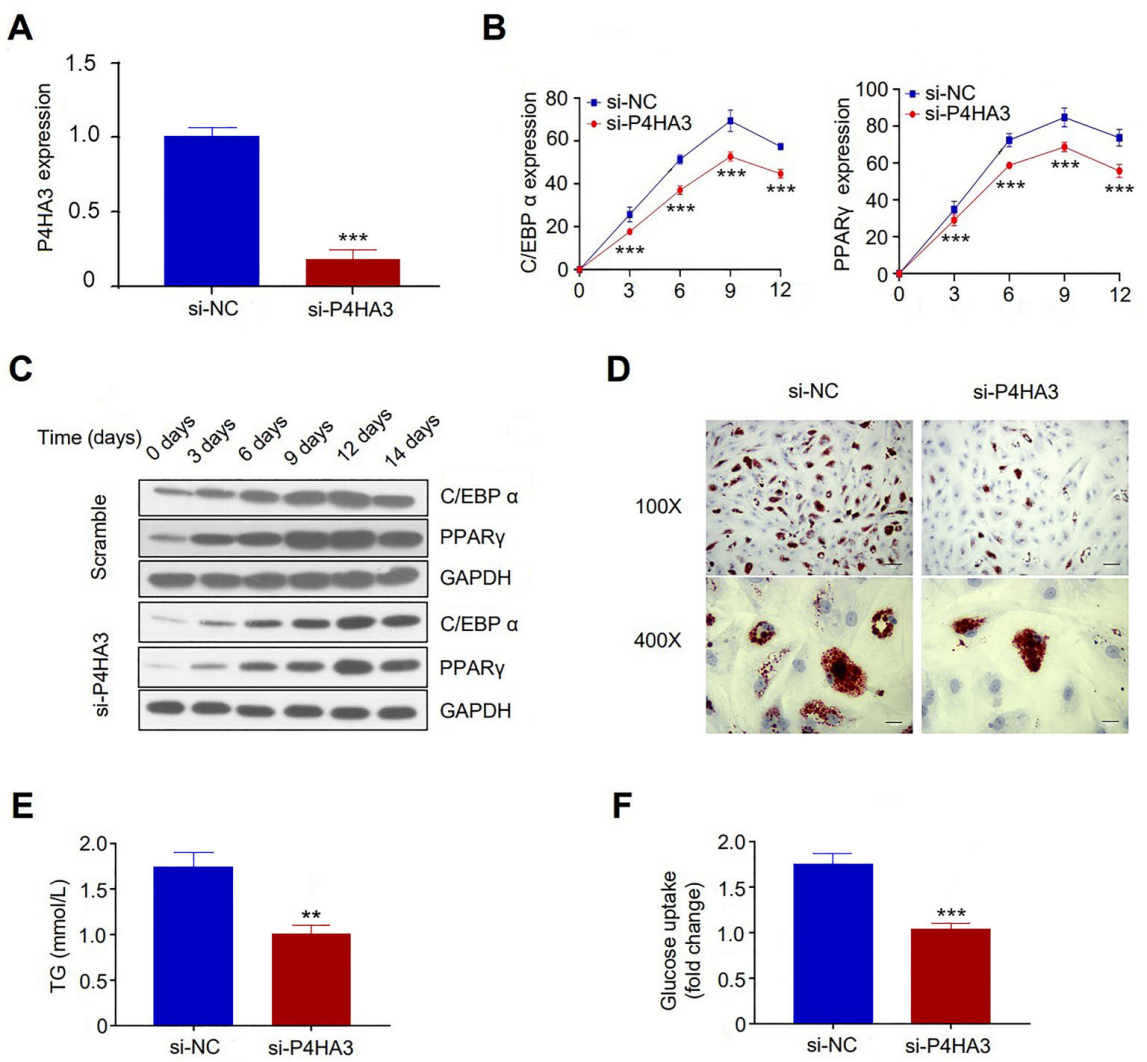
*P4HA3* knockdown inhibited adipocyte differentiation in 3T3-L1 cells. **A**, Expression of *P4HA3* following siRNA treatment was detected by qRT-PCR. **B** and **C**, *C/EBP-α* and *PPAR-γ* expression during the course of adipocytes differentiation was quantified using RT-qPCR and western blot assay after P4HA3 knockdown. **D**, Representative Oil Red O staining of differentiated adipocytes with or without *P4HA3* knockdown (scale bar, 20 μm). **E** and **F**, Triglycerides (TG) and glucose uptake determination in differentiated adipocytes with or without *P4HA3* knockdown. NC: negative control. Data are reported as means±SD. **P<0.01; ***P<0.001 (ANOVA and *t*-test)

To explore the functional role of *P4HA3* in adipocyte differentiation, we performed a knockdown experiment using siRNA targeting *P4HA3* in 3T3-L1 cell. Successful knockdown was confirmed using qRT-PCR, which showed that the transfection of *P4HA3* siRNA significantly reduced *P4HA3* expression in the si-P4HA3 group compared to the si-NC group (Figure 2A). We then performed adipocyte differentiation of the cells with or without *P4HA3* knockdown. During the adipocyte differentiation, the expression levels of adipocyte markers *C/EBP-α* and *PPAR-γ* gradually increased; however, *P4HA3* knockdown significantly attenuated their upregulation as quantified by qRT-PCR and western blot (Figure 2B and C). ORO staining further indicated that *P4HA3* knockdown suppressed lipid droplet accumulation in adipocyte differentiation (Figure 2D). In addition, *P4HA3* knockdown also lowered the triglyceride (TG) level and impaired the glucose uptake in the differentiated adipocytes (Figure 2E and F). Together, these data suggested that *P4HA3* knockdown suppressed adipocyte differentiation and insulin sensitivity in 3T3-L1 cells.

### *P4HA3* silencing counteracted HFD-induced obesity and improved insulin resistance in db/db mice

**Figure 3:**
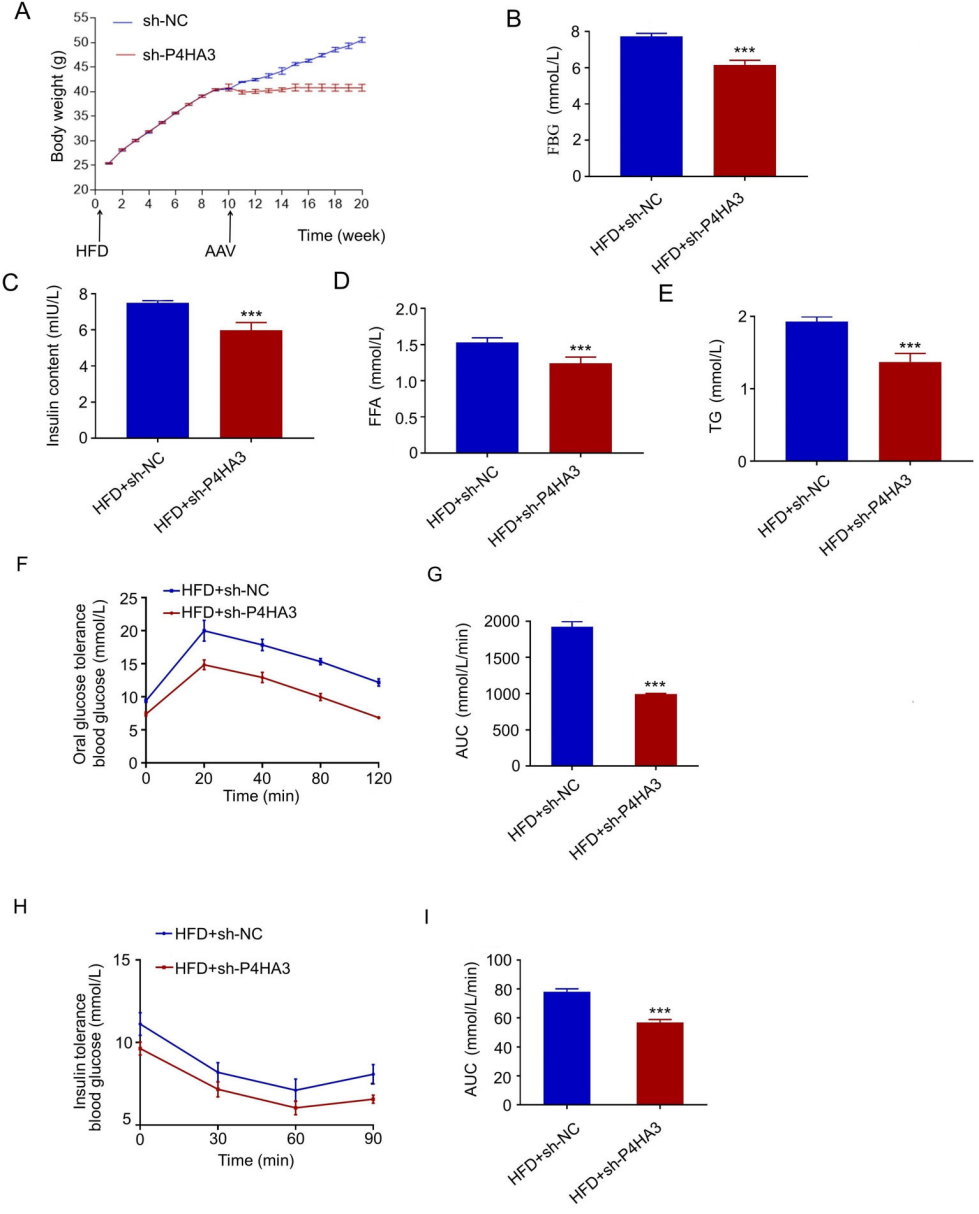
*P4HA3* knockdown counteracted high fat diet (HFD)-induced obesity and improved insulin resistance in db/db mice. **A**, C57BL6 db/db mice were fed with HFD, and then injected with AAV-shRNA-P4HA3 or AAV-scrambled shRNA. The body weight was monitored during the experiment. **B** and **C**, Fasting blood glucose (FBG) and insulin levels were determined after 6-h fasting at 20 weeks. **D** and **E**, Free fatty acid (FFA) and triglycerides (TG) levels in the blood were determined after 6-h fasting at 20 weeks. **F**, OGTT (oral glucose tolerance test) was performed at 20 weeks. **G**, Glucose AUC (area under the curve) was determined by OGTT. **H**, Insulin tolerance tests (ITT) was performed at 20 weeks. **I**, Insulin AUC was determined by ITTs. NC: negative control. Data are reported as means±SD. ***P<0.001 (*t*-test).

To investigate the functional role of *P4HA3* in the animal model, C57BL/6J db/db mice (2 months old) were fed with HFD for 10 weeks to induce obesity and diabetes. The mice were then randomly divided into sh-P4HA3 group and sh-NC (negative control) group (n=6/group). The mice were intraperitoneally injected with AAV9 vectors encoding an AAV-shRNA-P4HA3 sequence (shRNA targeting *P4HA3*) or AAV-scramble shRNA sequence. The mice in the sh-P4HA3 group did not show body weight increase after AAV injection (Figure 3A). Also, mice with *P4HA3* silencing had significantly lower fat mass and more lean mass (Supplementary Figure S1). In addition, after AAV9 vectors was administered (24 h after *P4HA3* knockdown), food intake in mice of the sh-P4HA3 group was significantly reduced compared with the sh-NC group (Supplementary Figure S2), which suggested that silencing *P4HA3* was beneficial for reducing food intake in mice. We also verified the knockdown of *P4HA3* in liver, epidermal adipose tissue, inguinal adipose tissue, skeletal muscle, islet, and hypothalamus in mice injected with AAV-shRNA-P4HA3 (Supplementary Figure S2). To examine the metabolic effect of *P4HA3* knockdown, FBG, insulin, free fatty acid (FFA), and TG levels were measured, and the results showed that *P4HA3* knockdown significantly reduced FGB, insulin, FFA, and TG levels in blood after 6-h fasting (Figure 3B-E). We also performed oral glucose tolerance test (OGTT) and insulin tolerance tests (ITT). The results demonstrated that *P4HA3* silencing increased oral glucose tolerance and improved insulin resistance in db/db mice (Figure 3F-I). Therefore, *P4HA3* silencing could potentially enhance insulin resistance and reduce obesity in db/db mice.

### *P4HA3* knockdown modulated adipocyte differentiation and insulin sensitivity in adipose tissue

**Figure 4:**
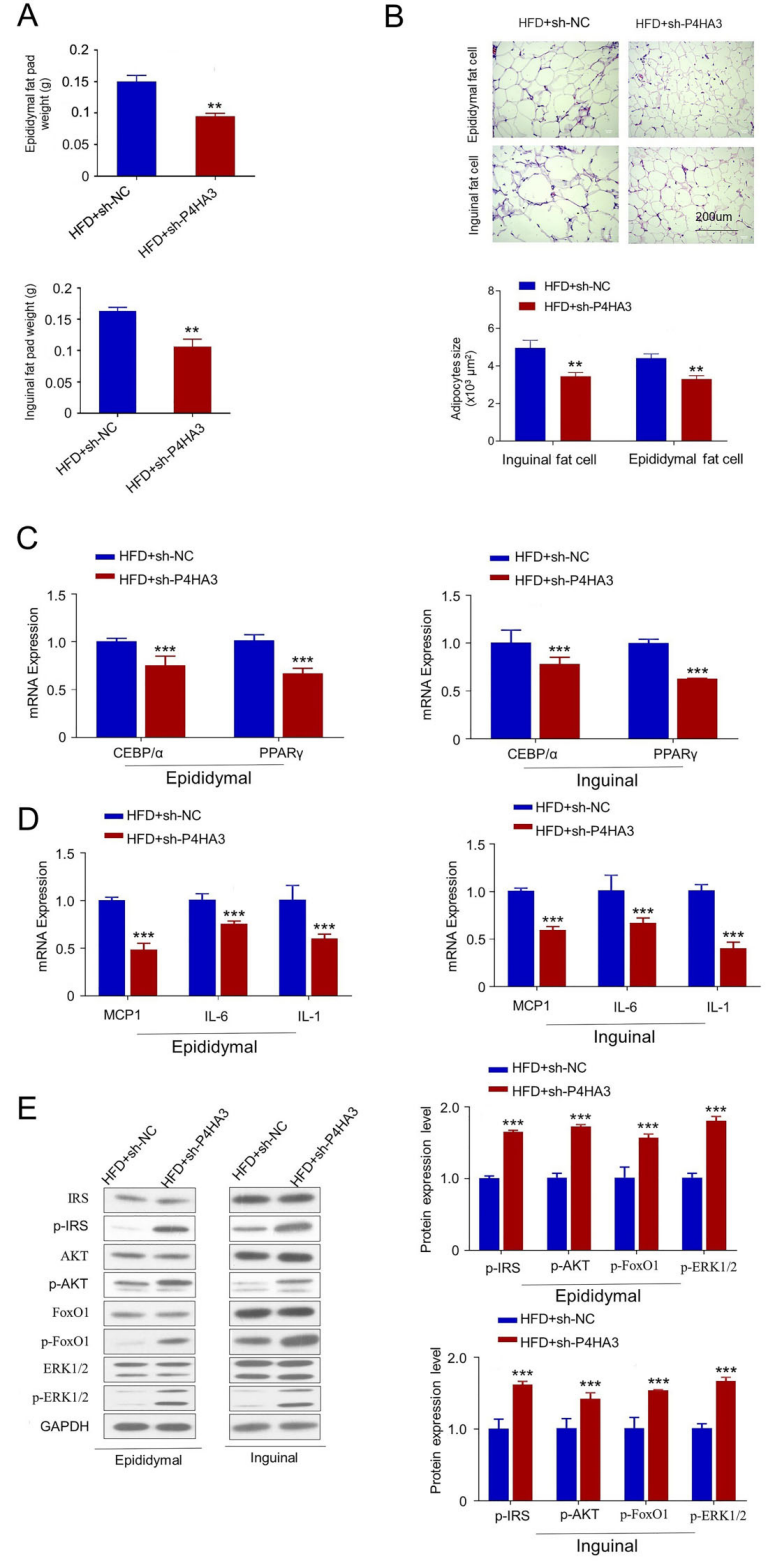
*P4HA3* silencing modulated adipocyte differentiation and insulin sensitivity gene expressions in adipose tissue. **A**, Epididymal and inguinal fat pad weight (g) of db/db mice were measured. **B**, The cross-sectional area of adipocytes in adipose tissue was analyzed by HE staining (scale bar, 200 μm). **C** and **D**, C/EBP-α, PPAR-γ, and inflammatory factors (MCP1, interleukin (IL)-6, and IL-1) expression in epididymal and inguinal adipose tissues of db/db mice with or without *P4HA3* knockdown was quantified using RT-qPCR. Mice were sacrificed after 6-h fasting for tissue collection. **E,** The phosphorylation levels of FoxO1, IRS, PI3K, and AKT in epididymal and inguinal adipose tissues of db/db mice were analyzed by western blotting. HFD: high fat diet; NC: negative control. Data are reported as means±SD. **P<0.01; ***P<0.001 (*t*-test).

Next, we examined the effect of *P4HA3* silencing on lipogenesis and insulin sensitivity in adipose tissues of the sh-P4HA3 and sh-NC groups. The total mass of epididymal and inguinal adipose tissues was significantly lower in the sh-P4AH3 group compared to that of the sh-NC group (Figure 4A). AAV-shRNA-P4HA3 injection also attenuated the increase of adipocyte cell size in adipose tissue (Figure 4B). Moreover, the expression of *CEBP/α* and *PPAR-γ* were significantly lower in both epididymal and inguinal adipose tissues of the sh-P4AH3 group compared to that of the sh-NC group (Figure 4C). Low-grade inflammation is a characteristic of T2DM (17). We therefore analyzed the expression of key inflammatory cytokines by RT-qPCR. The expression of these inflammatory genes such as *MCP1*, interleukin (*IL*)*-6*, and *IL-1* were significantly downregulated in the sh-P4HA3 group (Figure 4D). We also examined the insulin-related signaling proteins by western blot. We found that *P4HA3* knockdown significantly increased the phosphorylation level of IRS, Akt, FoxO1, and PI3K, which are key signaling proteins involved in insulin signaling transduction (Figure 4E). Together, these data suggested that *P4HA3* knockdown impaired adipocyte differentiation and insulin sensitivity in adipose tissue.

### *P4HA3* silencing improved hepatic glucose homeostasis and steatosis in db/db mice

**Figure 5:**
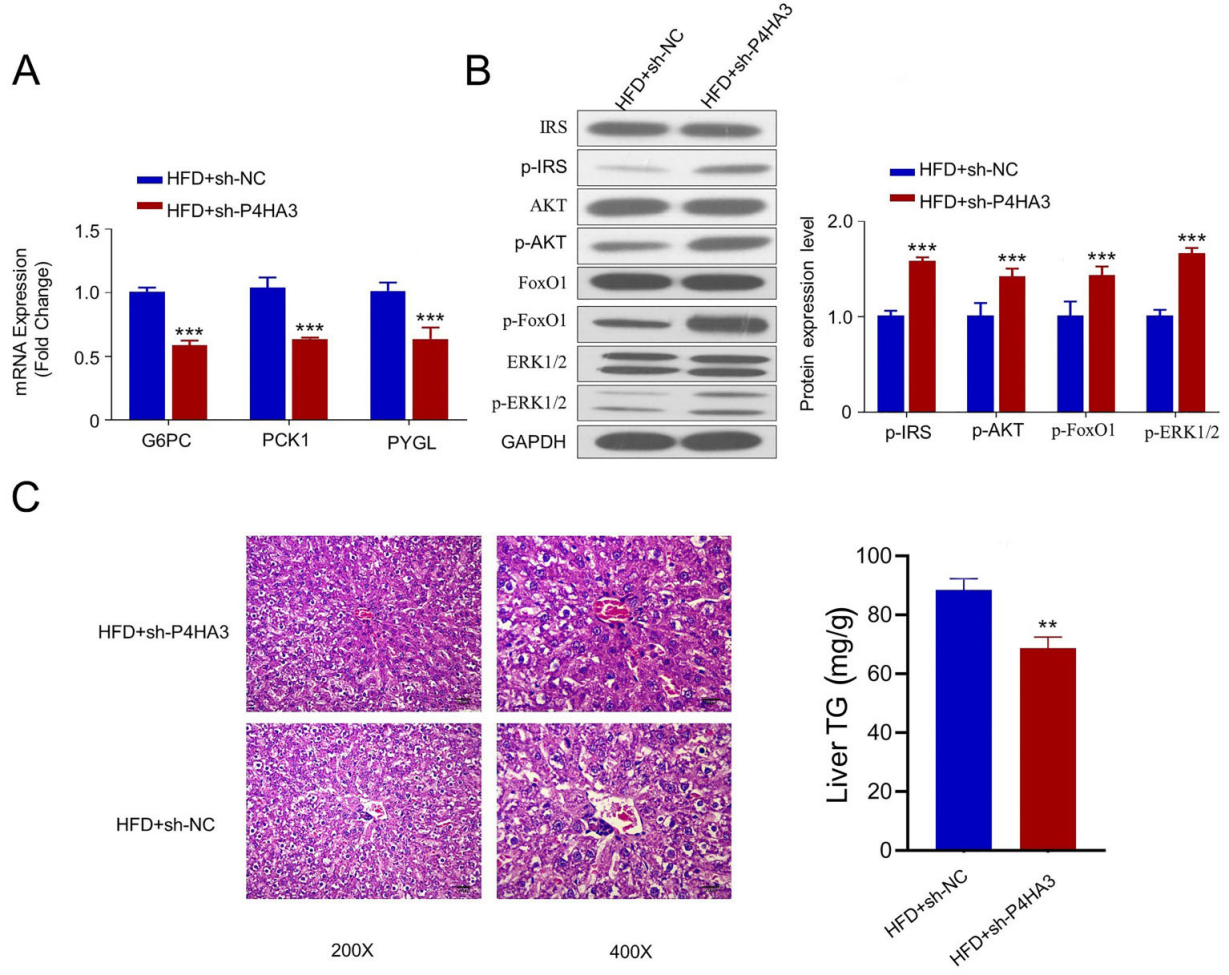
*P4HA3* improves hepatic glucose homeostasis and steatosis in db/db mice. **A**, The expression levels of gluconeogenesis genes (*G6PC* and *PCK1*) and glycogenolytic gene (*PYGL*) in liver tissues of db/db mice were determined by RT-qPCR. Mice were sacrificed after 6-h fasting for tissue collection. **B**, The phosphorylation levels of FoxO1, IRS, PI3K, and AKT in liver tissues of db/db mice with or without P4HA3 knockdown were analyzed using western blotting. **C**, Hepatic steatosis and liver triglycerides (TG) levels in db/db mice with or without *P4HA3* knockdown were examined by HE staining (scale bar, 20 μm) and TG kit. HFD: high fat diet; NC: negative control. Data are reported as means±SD. **P<0.01, ***P<0.001 (*t*-test).

To further validate the involvement of *P4HA3* in T2DM, hepatic glucose homeostasis, and steatosis in db/db mice were examined. Key genes involved in gluconeogenesis (*G6PC* and *PCK1*) and the glycogenolytic gene (*PYGL*) were significantly downregulated in the sh-P4HA3 group (Figure 5A). Also, the phosphorylation level of proteins related to insulin receptor signaling pathway were significantly enhanced in the sh-P4HA3 group (Figure 5B). In addition, H&E staining showed that the hepatic steatosis level in the sh-P4HA3 group was significantly reduced (Figure 5C). Together, these data suggested that *P4HA3* silencing improved hepatic glucose homeostasis and steatosis in db/db mouse model.

## Discussion

As a major risk factor for T2DM, obesity is characterized by a decreased response to insulin signaling pathways in multiple key tissues, such as adipose, liver, and muscle (18). However, obese patients do not necessarily suffer from T2DM, and many obese patients have a normal fasting blood glucose and insulin level (19). Recent study using mRNA profiling of adipose tissues has revealed differentially expressed genes between obese non-diabetic patients and obese patients with T2DM (20). These genes are mainly associated with insulin resistance or type 2 diabetes. We therefore analyzed published microarray data of adipocytes from non-obese controls, non-diabetic obese individuals, and obese individuals with diabetes. *P4HA3* was identified among the most profoundly upregulated genes in obese individuals or diabetic individuals, whose functional role in obesity and diabetes remain unknown.

It was reported that the dysregulation of P4H is associated with tumor initiation and progression, and P4H upregulation could augment the invasiveness potential of cancer cells and enhance the metastasis to lymph nodes and lungs (21). High expression of *P4HA3* seems correlated with dynamic extracellular matrix (ECM) remodeling and worse prognosis in breast cancer (22). However, the potential role of *P4HA3* in obesity and its association with T2DM are not well characterized. Our study revealed the upregulation of *P4HA3* in mature adipocytes from obese patients with T2DM. Further functional study demonstrated that the knockdown of *P4HA3* could reduce the mass of epididymal and inguinal adipose tissues and decrease the levels of fasting blood glucose, insulin, free fatty acid, and triglycerides in the obese mouse model. These results indicated that *P4HA3* upregulation may account for the dysregulation of adipocyte and insulin response in obesity combined with T2DM. Adipose tissue is a heterogeneous tissue responsible for systemic energy homeostasis (23). The susceptibility of obese patients to T2DM development can be attributed to the metabolic disorders caused by adipocytes (24). The excessive accumulation of adipose tissue can lead to dyslipidemia, adipocyte hypertrophy, and insulin resistance in obese individuals (25).

Through regulating temperature and calorie consumption, adipocytes play a pivotal role in maintaining homeostasis and energy balance. However, imbalanced dietary patterns or lack of exercise can lead to the fatty acid accumulation in adipocytes (26), which leads to abnormal adipocyte differentiation (27). PPARγ and CEBP/α are key transcription factors promoting adipogenic differentiation (28). In the present study, we demonstrated that *P4HA3* silencing could tune down the expression of PPAR-γ and CEBP-α. Our results also demonstrated that *P4HA3* knockdown could significantly decrease lipid accumulation and impair adipocyte differentiation in 3T3-L1 cells, suggesting that *P4HA3* was implicated in the regulation of adipocyte differentiation.

In the *in vivo* model, our work showed that *P4HA3* knockdown significantly affected systemic metabolism, including diet-induced obesity, insulin resistance, and liver steatosis. These changes can be attributed to the reduced food intake and enhanced glucose tolerance and homeostasis regulation. At the molecular level, *P4HA3* knockdown not only tunes down adipocyte differentiation, but it also downregulates the expression of inflammatory factors. Increased levels of inflammatory cytokines such as IL-1, IL-6, MCP-1, and leptin, and a decreased level of adiponectin are proposed to promote the development of insulin resistance and T2DM (29). IL-1, MCP-1, and IL-6 have been shown to impair insulin action in adipose tissue, liver, and skeletal muscle (30). We observed that the mRNA expression levels of IL-1, MCP-1, and IL-6 were significantly reduced in adipose tissue of mice with *P3HA4* silencing, which may be related to the improvement of insulin resistance induced by *P3HA4* silencing. It is also important to investigate the change of leptin and adiponectin in the future to get a full picture of how *P3HA4* silencing orchestrates adipocyte cytokine profiles.

IRS-1/PI3K/AKT signaling pathway is crucial in cell proliferation, differentiation, and adaptation, which also modulate the signal transduction initiated by insulin receptor (31). FoxO1, a member of the fork head family, is a main transcription factor downstream of the IRS-1/PI3K/Akt pathway (32). It has been reported that FoxO1 expression is increased in T2DM and FoxO1 could increase the transcription of the genes involved in gluconeogenesis (33). A previous study demonstrated that *P4HA3* can suppress the growth and metastasis of pituitary adenoma via blocking PI3K-Akt pathway (34). Consistently, our data showed that *P3HA3* knockdown can improve insulin resistance by activating IRS-1/PI3K/Akt/FoxO1 signaling pathway.

Liver is a central organ for carbohydrate metabolism via glycogenolysis and gluconeogenesis during fasting. The metabolic capacity is manifested as the expression level and activity of rate-limiting enzymes in glycogenolysis and gluconeogenesis (35). The increased hepatic glucose level could be attributed to the enhanced glycogenolysis or gluconeogenesis (35). Upon starvation, the expression of the genes encoding key rate-limiting enzymes, including PYGL, PCK1, and G6PC, are upregulated (36,37). The hyperglycemia observed in diabetes results from pancreatic dysfunction and insulin resistance, and is associated with unbalanced glycogenolysis and gluconeogenesis (38). Previous studies observed significant correlation between insulin resistance and PCK1, G6PC, and PYGL levels (39,40). In our results, we observed a lower expression of *PYGL*, *PCK1*, and *G6PC* in the liver of *P4HA3*-silenced obese mice. Since glycogenolysis and gluconeogenesis are primary drivers of hepatic glucose level, these data suggest that *P4HA3* could regulate hepatic glucose level by modulating genes involved in gluconeogenesis.

However, although the *in vivo P4HA3* silencing by intraperitoneal injection with AAV counteracts HFD-induced obesity and improves insulin resistance, we could not conclude whether the effects come directly from its influence in adipose tissues or liver tissues. Since we observed the downregulation of *P4HA3* expression in multiple tissues including liver and adipose tissues after AAV injection, we speculated that the protective effects may result from a systemic influence on multiple organs. Future work using tissue specific knockout mice will be needed to pinpoint the key tissues implicated in the *P4HA3*-dependent regulation. In addition, future studies will also need to fully investigate the expression and functional role of *P4HA3* between obese diabetic mice and lean mice.

In summary, this study demonstrated that *P4HA3* expression was increased in obese individuals with T2DM. We also provided evidence that *P4HA3* knockdown could ameliorate obesity and insulin resistance in C57BL/6J db/db mice with HFD feeding. *P4HA3* knockdown could also suppress adipocyte differentiation, alleviate the inflammatory cytokine expression, and improve the regulation of hepatic glucose homeostasis and steatosis. Our data suggested that targeting *P4HA3* may serve as a potential therapeutic strategy to ameliorate obesity-associated diabetes. Future work will be needed to delineate the mechanism by which *P4HA*3 becomes upregulated in obese and diabetic individuals.

## Supplementary Material

Click here to view [pdf].

## References

[1] Mrabti HN, Jaradat N, Kachmar MR, Ed-Dra A, Ouahbi A, Cherrah Y (2019). Integrative herbal treatments of diabetes in Beni Mellal region
of Morocco. J Integr Med.

[2] Okamoto Y, Kihara S, Funahashi T, Matsuzawa Y, Libby P (2006). Adiponectin: a key adipocytokine in metabolic
syndrome. Clin Sci (Lond).

[3] Yu X, Wang L, Zhang W, Ming J, Jia A, Xu S (2019). Fasting triglycerides and glucose index is more suitable for the
identification of metabolically unhealthy individuals in the Chinese adult
population: a nationwide study. J Diabetes Investig.

[4] Lawler HM, Underkofler CM, Kern PA, Erickson C, Bredbeck B, Rasouli N (2016). Adipose tissue hypoxia, inflammation, and fibrosis in obese
insulin-sensitive and obese insulin-resistant subjects. J Clin Endocrinol Metab.

[5] Mutch DM, Tordjman J, Pelloux V, Hanczar B, Henegar C, Poitou C (2009). Needle and surgical biopsy techniques differentially affect
adipose tissue gene expression profiles. Am J Clin Nutr.

[6] Bosello O, Donataccio MP, Cuzzolaro M (2016). Obesity or obesities? Controversies on the association between
body mass index and premature mortality. Eat Weight Disord.

[7] Cardona A, Day FR, Perry JRB, Loh M, Chu AY, Lehne B (2019). Epigenome-wide association study of incident type 2 diabetes in a
British population: EPIC-Norfolk Study. Diabetes.

[8] Zhuang XF, Zhao MM, Weng CL, Sun NL (2009). Adipocytokines: a bridge connecting obesity and insulin
resistance. Med Hypotheses.

[9] Atawia RT, Bunch KL, Toque HA, Caldwell RB, Caldwell RW (2019). Mechanisms of obesity-induced metabolic and vascular
dysfunctions. Front Biosci (Landmark Ed).

[10] Smith U, Kahn BB (2016). Adipose tissue regulates insulin sensitivity: role of
adipogenesis, de novo lipogenesis and novel lipids. J Intern Med.

[11] Hatzimichael E, Lo Nigro C, Lattanzio L, Syed N, Shah R, Dasoula A (2012). The collagen prolyl hydroxylases are novel transcriptionally
silenced genes in lymphoma. Br J Cancer.

[12] Wang Q, Zhang J, Fang S, Wang J, Han X, Liu F (2021). P4HA1 down-regulation inhibits glioma invasiveness by promoting
M1 microglia polarization. Onco Targets Ther.

[13] Xiong G, Deng L, Zhu J, Rychahou PG, Xu R (2014). Prolyl-4-hydroxylase α subunit 2 promotes breast cancer
progression and metastasis by regulating collagen deposition. BMC Cancer.

[14] Song H, Liu L, Song Z, Ren Y, Li C, Huo J (2018). P4HA3 is epigenetically activated by slug in gastric cancer and
its deregulation is associated with enhanced metastasis and poor
survival. Technol Cancer Res Treat.

[15] Li M, Wang Q, Zheng Q, Wu L, Zhao B, Wu Y (2021). Prognostic and diagnostic roles of prolyl 4-hydroxylase subunit α
members in breast cancer. Biomark Med.

[16] Kimura T, Ferran B, Tsukahara Y, Shang Q, Desai S, Fedoce A (2019). Production of adeno-associated virus vectors for *in
vitro* and *in vivo* applications. Sci Rep.

[17] Akash MS, Shen Q, Rehman K, Chen S (2012). Interleukin-1 receptor antagonist: a new therapy for type 2
diabetes mellitus. J Pharm Sci.

[18] Bastard JP, Maachi M, Lagathu C, Kim MJ, Caron M, Vidal H (2006). Recent advances in the relationship between obesity,
inflammation, and insulin resistance. Eur Cytokine Netw.

[19] Mikalsen SM, Bjørke-Monsen AL, Whist JE, Aaseth J (2019). Improved magnesium levels in morbidly obese diabetic and
non-diabetic patients after modest weight loss. Biol Trace Elem Res.

[20] Pinelli M, Giacchetti M, Acquaviva F, Cocozza S, Donnarumma G, Lapice E (2006). Beta2-adrenergic receptor and UCP3 variants modulate the
relationship between age and type 2 diabetes mellitus. BMC Med Genet.

[21] Gorres KL, Raines RT (2010). Prolyl 4-hydroxylase. Crit Rev Biochem Mol Biol.

[22] Winslow S, Lindquist KE, Edsjö A, Larsson C (2016). The expression pattern of matrix-producing tumor stroma is of
prognostic importance in breast cancer. BMC Cancer.

[23] Liu Z, Wu K, Jiang X, Xu A, Cheng K (2020). The role of adipose tissue senescence in obesity- and
ageing-related metabolic disorders. Clin Sci (Lond).

[24] Zhen Q, Yao N, Chen X, Zhang X, Wang Z, Ge Q (2019). Total body adiposity, triglycerides, and leg fat are independent
risk factors for diabetic peripheral neuropathy in Chinese patients with
type 2 diabetes mellitus. Endocr Pract.

[25] de Souza CJ, Eckhardt M, Gagen K, Dong M, Chen W, Laurent D (2001). Effects of pioglitazone on adipose tissue remodeling within the
setting of obesity and insulin resistance. Diabetes.

[26] Kahn CR, Wang G, Lee KY (2019). Altered adipose tissue and adipocyte function in the pathogenesis
of metabolic syndrome. J Clin Invest.

[27] Ghaben AL, Scherer PE (2019). Adipogenesis and metabolic health. Nat Rev Mol Cell Biol.

[28] Zhang W, Cline MA, Liu D, Gilbert ER (2013). Knockdown of ZBED6 is not associated with changes in murine
preadipocyte proliferation or differentiation. Adipocyte.

[29] Forny-Germano L, De Felice FG, Vieira MNN (2018). The role of leptin and adiponectin in obesity-associated
cognitive decline and Alzheimer's disease. Front Neurosci.

[30] Mitrou P, Raptis SA, Dimitriadis G (2013). Insulin action in morbid obesity: a focus on muscle and adipose
tissue. Hormones (Athens).

[31] Farrar C, Houser CR, Clarke S (2005). Activation of the PI3K/Akt signal transduction pathway and
increased levels of insulin receptor in protein repair-deficient
mice. Aging Cell.

[32] Smerieri A, Montanini L, Maiuri L, Bernasconi S, Street ME (2014). FOXO1 content is reduced in cystic fibrosis and increases with
IGF-I treatment. Int J Mol Sci.

[33] Wu Y, Pan Q, Yan H, Zhang K, Guo X, Xu Z (2018). Novel mechanism of Foxo1 phosphorylation in glucagon signaling in
control of glucose homeostasis. Diabetes.

[34] Long R, Liu Z, Li J, Yu H (2019). COL6A6 interacted with P4HA3 to suppress the growth and
metastasis of pituitary adenoma via blocking PI3K-Akt
pathway. Aging (Albany NY).

[35] Oh KJ, Han HS, Kim MJ, Koo SH (2013). Transcriptional regulators of hepatic
gluconeogenesis. Arch Pharm Res.

[36] Hatting M, Tavares C, Sharabi K, Rines AK, Puigserver P (2018). Insulin regulation of gluconeogenesis. Ann N Y Acad Sci.

[37] Davit-Spraul A, Piraud M, Dobbelaere D, Valayannopoulos V, Labrune P, Habes D (2011). Liver glycogen storage diseases due to phosphorylase system
deficiencies: diagnosis thanks to non invasive blood enzymatic and molecular
studies. Mol Genet Metab.

[38] Wise S, Nielsen M, Rizza R (1997). Effects of hepatic glycogen content on hepatic insulin action in
humans: alteration in the relative contributions of glycogenolysis and
gluconeogenesis to endogenous glucose production. J Clin Endocrinol Metab.

[39] Villa-Pérez P, Merino B, Fernández-Díaz CM, Cidad P, Lobatón CD, Moreno A (2018). Liver-specific ablation of insulin-degrading enzyme causes
hepatic insulin resistance and glucose intolerance, without affecting
insulin clearance in mice. Metabolism.

[40] Stygar D, Andrare D, Bażanów B, Chełmecka E, Sawczyn T, Skrzep-Poloczek B (2019). The Impact of DJOS surgery, a high fat diet and a control diet on
the enzymes of glucose metabolism in the liver and muscles of Sprague-Dawley
rats. Front Physiol.

